# Acknowledgement to reviewers

**DOI:** 10.1186/s40792-016-0138-5

**Published:** 2016-02-12

**Authors:** Hideo Baba

**Affiliations:** Department of Gastroenterological Surgery, Graduate School of Medical Sciences, Kumamoto University, Kumamoto, Japan

## Abstract

The Editorial Board of *Surgical Case Reports* expresses their appreciation to the following consultants for reviewing manuscripts during 2014–2015.

**Ken Aiyama**

Japan

**Toshiaki Akita**

Japan

**Hironobu Amano**

Japan

**Tohru Asai**

Japan

**Takayuki Asao**

Japan

**Nobuyoshi Azuma**

Japan

**Toru Beppu**

Japan

**Masayuki Chida**

Japan

**Takashi Chishima**

Japan

**Haruhiko Cho**

Japan

**Hiroshi Date**

Japan

**Hiroyoshi Doihara**

Japan

**Yuichiro Doki**

Japan

**Akinori Egashira**

Japan

**Hiroyuki Egi**

Japan

**Susumu Eguchi**

Japan

**Shunsuke Endo**

Japan

**Itaru Endo**

Japan

**Hideki Fujii**

Japan

**Jiro Fujimoto**

Japan

**Hikaru Fujioka**

Japan

**Fumihiko Fujita**

Japan

**Toshiyoshi Fujiwara**

Japan

**Minoru Fukuchi**

Japan

**Ikuo Fukuda**

Japan

**Takumi Fukumoto**

Japan

**Ryoji Fukushima**

Japan

**Kazuhito Funai**

Japan

**Tomohisa Furuhata**

Japan

**Ryoichi Goto**

Japan

**Kenichi Hakamada**

Japan

**Madoka Hamada**

Japan

**Kazuhiro Hanazaki**

Japan

**Kazuo Hase**

Japan

**Seiki Hasegawa**

Japan

**Kazuhiro Hashimoto**

Japan

**Makoto Hashizume**

Japan

**Shoichi Hazama**

Japan

**Yasuhiro Hida**

Japan

**Takao Hinoi**

Japan

**Kosei Hirakawa**

Japan

**Satoshi Hirano**

Japan

**Shoichi Hishinuma**

Japan

**Shigenori Homma**

Japan

**Jun Horiguchi**

Japan

**Akihiko Horiguchi**

Japan

**Yasushi Ichikawa**

Japan

**Satoshi Ida**

Japan

**Hitoshi Idani**

Japan

**Satoshi Ieiri**

Japan

**Akio Ikai**

Japan

**Hitoshi Ikeda**

Japan

**Tadashi Ikeda**

Japan

**Norihiko Ikeda**

Japan

**Toru Ikegami**

Japan

**Masahide Ikeguchi**

Japan

**Hiroki Ikeuchi**

Japan

**Yutaka Imoto**

Japan

**Shigeru Imoto**

Japan

**Masayoshi Inoue**

Japan

**Shuji Isaji**

Japan

**Hiroyuki Ishibashi**

Japan

**Hideyuki Ishida**

Japan

**Fumio Ishida**

Japan

**Tetsuro Ishikawa**

Japan

**Yoh Isobe**

Japan

**Kenichi Ito**

Japan

**Koji Ito**

Japan

**Akinori Iwasaki**

Japan

**Hirotaka Iwase**

Japan

**Hisashi Iwata**

Japan

**Yuichi Izumi**

Japan

**Mitsutaka Kadokura**

Japan

**Yoshihisa Kadota**

Japan

**Kichizo Kaga**

Japan

**Takashi Kamei**

Japan

**Hiroyuki Kamiya**

Japan

**Toshiya Kamiyama**

Japan

**Toshio Kanai**

Japan

**Koichi Kaneko**

Japan

**Nobuyasu Kano**

Japan

**Natsuya Katada**

Japan

**Takahiro Katsumata**

Japan

**Naoki Kawagishi**

Japan

**Masafumi Kawamura**

Japan

**Tatsuyuki Kawano**

Japan

**Shiro Kikuchi**

Japan

**Yasue Kimura**

Japan

**Takahiro Kinoshita**

Japan

**Yoshiaki Kinoshita**

Japan

**Tetsuya Kitagawa**

Japan

**Hiroaki Kitagawa**

Japan

**Hirokazu Kiyozaki**

Japan

**Akira Kobayashi**

Japan

**Tsuyoshi Kobayashi**

Japan

**Keiji Koda**

Japan

**Akio Kodama**

Japan

**Yasuhiro Kodera**

Japan

**Hiroyoshi Komai**

Japan

**Kimihiro Komori**

Japan

**Masanori Kon**

Japan

**Kazuya Kondo**

Japan

**Takeo Kosaka**

Japan

**Chihiro Kosugi**

Japan

**Yonson Ku**

Japan

**Shoji Kubo**

Japan

**Kiyoshi Kubochi**

Japan

**Keiichi Kubota**

Japan

**Chikara Kunisaki**

Japan

**Nobuhiro Kurita**

Japan

**Shintaro Kuroda**

Japan

**Katsumasa Kuroi**

Japan

**Tamotsu Kuroki**

Japan

**Masato Kusunoki**

Japan

**Hiroyuki Kuwano**

Japan

**Yoshihiko Maehara**

Japan

**Yoshimasa Maniwa**

Japan

**Munetaka Masuda**

Japan

**Junichi Matsui**

Japan

**Isao Matsumoto**

Japan

**Naoki Matsumura**

Japan

**Akira Mishima**

Japan

**Tetsuya Mitsudomi**

Japan

**Norio Mitsumori**

Japan

**Shinichi Miyagawa**

Japan

**Hidenori Miyake**

Japan

**Shinji Miyamoto**

Japan

**Hidetoshi Miyauchi**

Japan

**Tatsuya Miyazaki**

Japan

**Yukimasa Miyazawa**

Japan

**Toru Mizuguchi**

Japan

**Masaru Mizuno**

Japan

**Akira Mogi**

Japan

**Shinichiro Mori**

Japan

**Toshiaki Morikawa**

Japan

**Masaru Morita**

Japan

**Fuyuhiko Motoi**

Japan

**Satoru Motoyama**

Japan

**Yoshiaki Murakami**

Japan

**Soichiro Murata**

Japan

**Hiroaki Musha**

Japan

**Takeshi Nagayasu**

Japan

**Takeshi Naitoh**

Japan

**Koji Nakagawa**

Japan

**Kei Nakagawa**

Japan

**Toshio Nakagohri**

Japan

**Masanobu Nakajima**

Japan

**Yoshiyuki Nakajima**

Japan

**Jun Nakajima**

Japan

**Hiroshige Nakamura**

Japan

**Masafumi Nakamura**

Japan

**Masato Nakamura**

Japan

**Kanyu Nakano**

Japan

**Yuichiro Nakashima**

Japan

**Haruhiko Nakayama**

Japan

**Mitsuo Nakayama**

Japan

**Kazutaka Narui**

Japan

**Shoji Natsugoe**

Japan

**Riichiro Nezu**

Japan

**Itasu Ninomiya**

Japan

**Motonobu Nishimura**

Japan

**Atsushi Nishimura**

Japan

**Tomohiro Nishinaka**

Japan

**Hiroyuki Nitta**

Japan

**Hirokazu Noshiro**

Japan

**Tomoko Ogawa**

Japan

**Hitoshi Ogino**

Japan

**Kei Ohara**

Japan

**Kippei Ohgaki**

Japan

**Masaichi Ohira**

Japan

**Takao Ohki**

Japan

**Nobuhiro Ohkohchi**

Japan

**Shinobu Ohnuma**

Japan

**Mitsuhiko Ohta**

Japan

**Norifumi Ohtani**

Japan

**Masayuki Ohtsuka**

Japan

**Kenoki Ohuchida**

Japan

**Hitoshi Ojima**

Japan

**Kan Okabayashi**

Japan

**Kazunori Okabe**

Japan

**Kenji Okada**

Japan

**Tatsuro Okamoto**

Japan

**Tomoyoshi Okamoto**

Japan

**Shinichi Okazumi**

Japan

**Yutaka Okita**

Japan

**Kenichi Okubo**

Japan

**Hiroshi Okumura**

Japan

**Kiyotaka Okuno**

Japan

**Mitsugu Omasa**

Japan

**Nobuo Omura**

Japan

**Shigeru Ono**

Japan

**Kazumasa Orihashi**

Japan

**Akihiko Oshita**

Japan

**Mitsuyoshi Ota**

Japan

**Takehito Otsubo**

Japan

**Toshiki Rikiyama**

Japan

**Yasushi Rino**

Japan

**Yoshihisa Saida**

Japan

**Yoshiharu Sakai**

Japan

**Shinichi Sakuramoto**

Japan

**Hironori Samura**

Japan

**Akihiko Sano**

Japan

**Takeo Sato**

Japan

**Yukio Sato**

Japan

**Masato Sato**

Japan

**Yukitoshi Satoh**

Japan

**Hiroyuki Satokawa**

Japan

**Noriyoshi Sawabata**

Japan

**Mitsugu Sekimoto**

Japan

**Takafumi Sekino**

Japan

**Yasuyuki Seto**

Japan

**Haruhiko Shida**

Japan

**Norihiko Shiiya**

Japan

**Hideaki Shimada**

Japan

**Jiro Shimazaki**

Japan

**Hiroaki Shimizu**

Japan

**Daisuke Shimizu**

Japan

**Hideto Shimpo**

Japan

**Hiroyuki Shinchi**

Japan

**Shunya Shindo**

Japan

**Satoshi Shiono**

Japan

**Shigehiro Shiozaki**

Japan

**Ken Shirabe**

Japan

**Yasuhiro Shirakawa**

Japan

**Masayuki Sho**

Japan

**Kiyohiko Shuto**

Japan

**Makoto Sohda**

Japan

**Hideto Sonoda**

Japan

**Kenji Sugio**

Japan

**Masahiko Sugiyama**

Japan

**Masanori Sugiyama**

Japan

**Yasuo Sumi**

Japan

**Yasuyuki Suzuki**

Japan

**Makoto Suzuki**

Japan

**Tomoaki Taguchi**

Japan

**Hiroyuki Tahara**

Japan

**Yoshitsugu Tajima**

Japan

**Tatsuro Tajiri**

Japan

**Yasutsugu Takada**

Japan

**Takao Takahashi**

Japan

**Seiichi Takenoshita**

Japan

**Akinobu Taketomi**

Japan

**Hiroshi Takeyama**

Japan

**Yoshifumi Takeyama**

Japan

**Katsunari Takifuji**

Japan

**Hiroyuki Tanaka**

Japan

**Kuniya Tanaka**

Japan

**Kazuo Tanemoto**

Japan

**Akira Tangoku**

Japan

**Shigeki Taniguchi**

Japan

**Hirotaka Tashiro**

Japan

**Tadashi Tashiro**

Japan

**Yuko Tazuke**

Japan

**Masahiro Tominaga**

Japan

**Naohiro Tomita**

Japan

**Takeo Toshima**

Japan

**Yasuhiro Tsubosa**

Japan

**Masanori Tsuchida**

Japan

**Soichi Tsutsumi**

Japan

**Eiji Uchida**

Japan

**Hiroo Uchida**

Japan

**Kazuhisa Uchiyama**

Japan

**Toshihiko Ueda**

Japan

**Kenichiro Uemura**

Japan

**Sadashige Uemura**

Japan

**Nozomi Ueno**

Japan

**Masaki Ueno**

Japan

**Katsuhiko Uesaka**

Japan

**Naoto Urushihara**

Japan

**Akihiko Usui**

Japan

**Toshifumi Wakai**

Japan

**Yuji Watanabe**

Japan

**Yoshinori Watanabe**

Japan

**Minoru Yagi**

Japan

**Hitoshi Yaku**

Japan

**Masaaki Yamagishi**

Japan

**Akio Yamaguchi**

Japan

**Satoru Yamaguchi**

Japan

**Koji Yamaguchi**

Japan

**Yuzo Yamamoto**

Japan

**Manabu Yamamto**

Japan

**Hiroko Yamashita**

Japan

**Tomoki Yamatsuji**

Japan

**Hiroki Yamaue**

Japan

**Katsuhiko Yanaga**

Japan

**Motoki Yano**

Japan

**Kenji Yano**

Japan

**Tokujiro Yano**

Japan

**Takashi Yao**

Japan

**Seiei Yasuda**

Japan

**Takushi Yasuda**

Japan

**Kohei Yokoi**

Japan

**Hideki Yokoo**

Japan

**Takeo Yonekura**

Japan

**Kazuhiro Yoshida**

Japan

**Hiroshi Yoshida**

Japan

**Takaki Yoshikawa**

Japan

**Norio Yoshimura**

Japan

**Naoki Yoshimura**

Japan

**Ichiro Yoshino**

Japan

**Kazuhiko Yoshioka**

Japan

